# Evaluation of a Fast Protocol for Staging Lymphoma Patients with Integrated PET/MRI

**DOI:** 10.1371/journal.pone.0157880

**Published:** 2016-06-21

**Authors:** Johannes Grueneisen, Lino Morris Sawicki, Benedikt Michael Schaarschmidt, Saravanabavaan Suntharalingam, Sara von der Ropp, Axel Wetter, Verena Ruhlmann, Harald H. Quick, Michael Forsting, Lale Umutlu

**Affiliations:** 1 Department of Diagnostic and Interventional Radiology and Neuroradiology, University Hospital Essen, University of Duisburg-Essen, Essen, Germany; 2 Department of Diagnostic and Interventional Radiology, University Hospital Dusseldorf, University of Dusseldorf, Dusseldorf, Germany; 3 Department of Nuclear Medicine, University Hospital Essen, University of Duisburg-Essen, Essen, Germany; 4 Erwin L. Hahn Institute for Magnetic Resonance Imaging, University of Duisburg-Essen, Essen, Germany; 5 High Field and Hybrid MR Imaging, University Hospital Essen, University of Duisburg-Essen, Essen, Germany; University of Graz, AUSTRIA

## Abstract

**Background:**

The aim of this study was to assess the applicability of a fast MR-protocol for whole-body staging of lymphoma patients using an integrated PET/MR system.

**Methods:**

A total of 48 consecutive lymphoma patients underwent 52 clinically indicated PET/CT and subsequent PET/MRI examinations with the use of 18F-FDG. For PET/MR imaging, a fast whole-body MR-protocol was implemented. A radiologist and a nuclear medicine physician interpreted MRI and PET/MRI datasets in consensus and were instructed to identify manifestations of lymphoma on a site-specific analysis. The accuracy for the identification of active lymphoma disease was calculated and the tumor stage for each examination was determined. Furthermore, radiation doses derived from administered tracer activities and CT protocol parameters were estimated and the mean scan duration of PET/CT and PET/MR imaging was determined. Statistical analysis was performed to compare the diagnostic performance of PET/MRI and MRI alone. The results of PET/CT imaging, all available histopathological samples as well as results of prior examinations and follow-up imaging were used for the determination of the reference standard.

**Results:**

Active lymphoma disease was present in 28/52 examinations. PET/MRI revealed higher values of diagnostic accuracy for the identification of active lymphoma disease in those 52 examinations in comparison to MRI, however, results of the two ratings did not differ significantly. On a site specific analysis, PET/MRI showed a significantly higher accuracy for the identification of nodal manifestation of lymphoma (p<0.05) if compared to MRI, whereas ratings for extranodal regions did not reveal a significant difference. In addition, PET/MRI enabled correct identification of lymphoma stage in a higher percentage of patients than MRI (94% vs. 83%). Furthermore, SUVs derived from PET/MRI were significantly higher than in PET/CT, however, there was a strong positive correlation between SUVmax and SUVmean of the two imaging modalities (R = 0.91 p<0.001 and R = 0.87, p<0.001). Average scan duration of whole-body PET/CT and PET/MRI examinations amounted to 17.3±1.9 min and 27.8±3.7 min, respectively. Estimated mean effective-dose for whole-body PET/CT scans were 64.4% higher than for PET/MRI.

**Conclusions:**

Our results demonstrate the usefulness of 18F-FDG PET data as a valuable additive to MRI for a more accurate evaluation of patients with lymphomas. With regard to patient comfort related to scan duration and a markedly reduced radiation exposure, fast PET/MRI may serve as a powerful alternative to PET/CT for a diagnostic workup of lymphoma patients.

## Introduction

Highly accurate staging of lymphoma patients is mandatory to identify tumor localizations as well as disease extent, which provides important prognostic information and helps to select an appropriate treatment strategy, primarily based on chemotherapy and/or radiotherapy.

The use of diagnostic imaging has been shown a valuable tool for the determination of the initial tumor stage and to evaluate therapy response [1, 2]. Computed tomography (CT) is the most commonly applied method for a diagnostic workup of lymphoma patients, due to its high availability and the opportunity of rapid data collection. However, the successful introduction of hybrid imaging, in form of positron emission tomography/computed tomography (PET/CT), has been demonstrated to enable a more accurate clinical evaluation of the majority of lymphoma types [3, 4]. The additional metabolic information provided by 18F-Fluorodeoxyglucose (18F-FDG)-PET led to an improved staging performance as well as therapy response assessment based on changes in 18F-FDG uptake between baseline and interim or post-treatment scans [5–7]. While the combined information of PET/CT has been shown beneficial compared to other cross-sectional imaging techniques for the diagnostic workup of lymphoma patients, one major disadvantage is caused by an increased ionizing radiation dose due to the combination of PET and whole-body CT [8–10].

Initial studies on integrated positron emission tomography/magnetic resonance imaging (PET/MRI), combining the diagnostic advantages of simultaneous acquired PET and MRI data, have also shown promising results for the evaluation of patients with lymphoma [11, 12]. While PET/MRI compared to PET/CT offers the inherent advantage of reduced radiation dose, the interchange of the morphological part from CT to MRI as a part of hybrid imaging, has been shown to result in a markedly prolonged examination time [10], potentially resulting in patient discomfort.

Therefore, the aim of the present study was to investigate the diagnostic applicability of a fast protocol for whole-body staging lymphoma patients with simultaneous PET/MRI.

## Materials and Methods

### Patients

The study was conducted in conformance with the Declaration of Helsinki and approved by the Ethics Commission of the Medical Faculty of the University Duisburg-Essen (study number 11–4822-BO). Written informed consent was obtained from all patients before each examination. A total of 48 consecutive lymphoma patients (mean age 47±16 years; range 19–73 years) were prospectively enrolled in this trial. A total of 52 examinations were performed including scans for initial staging (n = 11), interim scans during treatment (n = 7), for restaging after the end of treatment (n = 9) and for surveillance/exclusion of tumor relapse upon suspicion (n = 25). Table 1 shows the distribution of the different lymphoma subtypes.

**Table 1:** Distribution of patients according to their diagnosis.

Lymphoma subtypes	No. of patients
Hodgkin’s disease	18
Diffuse large B-cell lymphoma	20
Follicular lymphoma	7
Peripheral T-cell lymphoma	2
MALT-lymphoma	1

### PET/CT

PET/CT scans were performed on a Biograph mCT 128 system (Siemens, Healthcare GmbH, Germany), after a fasting period of at least 6 hours. Prior to each examination, blood samples were taken to ensure blood glucose levels below 150 mg/dl. Then, a body-weight adapted dosage (4 MBq/kg bodyweight) of 18F-FDG, with a mean activity of 273±52 MBq, was intravenously administered 61±14 min before the start of each scan. Patients were examined in full-dose (n = 24) or low-dose (n = 28) technique. Whole-body CT examinations were performed in caudo-cranial scan direction with an increment of 5 mm and a pitch of 1, using a manufacturer-supplied dose reduction software for automatic mA/s adjustment (Care Dose 4D^™^, presets: full-dose: 120 kV, 210 mAs; low-dose: 120 kV, 40 mAs). Images were reconstructed with a slice thickness of 5 mm. Full-dose PET/CT scans started 70 s after intravenous administration of 100 ml of iodinated contrast media (*Ultravist 300*, Bayer Healthcare, Germany). PET data were obtained in 5–7 bed positions (from skull-base to upper thighs) with an acquisition time of 2 min each, a 256x256 matrix and a Gaussian filter of 4 mm Full Width at Half Maximum (FWHM). An attenuation weighted ordered-subset expectation maximization algorithm (AW-OSEM) was used for PET image reconstruction with 3 iterations and 24 subsets. Maps for attenuation correction were calculated based on acquired CT datasets. For estimations of the effective dose of whole-body CT scans (low-dose and full-dose), the dose-length product and a conversion factor were used as described in a previous study [13]. In accordance with a previous report, mean effective dose of PET was calculated based on the administered 18F-FDG dose [14].

### PET/MRI

PET/MRI scans were performed on a 3 Tesla Biograph mMR integrated PET/MR system (Siemens Healthcare GmbH, Germany). Patients were bedded head-first in supine position. Imaging started with an average delay of 133±25 min after the injection of 18F-FDG. Whole-body PET data were obtained in 4–5 bed positions (from skull-base to mid-thighs) with an acquisition time of 4 min each. PET image reconstruction was performed subsequently by the use of the OSEM algorithm, 3 iterations and 21 subsets, a Gaussian filter with 4 mm, FWHM and a 344x344 image matrix. PET datasets were automatically attenuation corrected using a four-compartment-model attenuation map (μ-map), calculated from fat-only and water-only datasets, as obtained by Dixon-based sequences. For MRI data acquisition a dedicated mMR head-and-neck radiofrequency (RF) coil and RF body array surface coils were used [15]. A fast protocol was implemented for MR imaging, using the following sequences: coronal 3-dimensional volume interpolated breath-hold examination (VIBE) sequence for Dixon-based attenuation correction (repetition time [TR], 3.6 ms; echo time [TE], 1.23 and 2.46 ms; 3.12 mm slice thickness; matrix size 192x79; FOV, 500 x 328 mm; acquisition time [TA], 19 sec); transversal diffusion-weighted (DWI) echo-planar imaging (EPI) sequence (TR, 9900 ms; TE, 82 ms; b-values: 0, 500 and 1000 s/mm^2^, 5.0 mm slice thickness; matrix size 160x90; FOV, 420 x 315 mm; TA, 2.48 min); transversal 2-dimensional half Fourier acquisition single-shot turbo spin echo (HASTE) sequence (TR, 1500 ms; TE, 117 ms; 5.0 mm slice thickness; matrix size 320x211; FOV, 450 x 366 mm; TA, 1.06 min), transversal post-contrast 3-dimensional fat-saturated VIBE sequence (TR, 4.08 ms; TE, 1.51 ms; 3.5 mm slice thickness, matrix size 512x230; FOV, 400 x 280 mm; TA, 18 sec). For contrast-enhanced imaging, 0.1 mmol/kg of Gadobutrol (Gadovist, Bayer HealthCare, Germany) was injected intravenously, followed by a saline flush of 20 ml/s using an automated injector (Spectris Solaris EP MR Injection System, Medrad, Germany).

### Image interpretation

Images were analysed by two board-certified physicians (radiologist, 8 years of experience; nuclear medicine physician (7 years of experience), in consensus and in random order, using a dedicated software for hybrid imaging (Syngo.via; Siemens, Healthcare GmbH, Germany). Both readers were blinded to the patients’ identification data. A first session comprised interpretation of MRI datasets followed by readings of PET/MRI data. An interval of 4 weeks between the ratings was chosen to avoid recognition bias. For each rating, the readers were instructed to identify manifestations of lymphoma on a site-specific analysis: nodal groups included Waldeyer ring, right and left cervical, right and left axillary, right and left internal mammary or diaphragmatic, anterior mediastinal or paratracheal, right and left hilar, subcarinal or posterior mediastinal, celiac or superior mesenteric, hepatic and splenic hilar, retroperitoneal, inferior mesenteric, right and left iliac and right and left inguinal regions. In addition, several extra nodal regions were analyzed, including lungs, liver, spleen, kidneys, thyroid, adrenal glands, bones, stomach, intestines as well as other different organs and tissues.

For all identified lesions size measurements were performed and the standardized uptake value (SUV) in PET positive lesions was determined by drawing a 3D-isocontour on fused PET/CT and PET/MR images. Furthermore, for both imaging modalities the mean scan duration was measured. Using DWI as a part of MR imaging, an ADC map was generated by the PET/MR system software (syngo VB18P, Siemens Healthcare GmbH, Germany) using three b-values (b = 0, 500 and 1000 s/mm^2^).

Malignancy on MRI was defined according the following criteria: nodal lesions with a longest diameter >1.5 cm and extra nodal masses with a longest diameter >1 cm, distinctive contrast enhancement, central necrosis, local tumor invasion/destruction, high signal intensity in DWI (b = 1000 s/mm^2^) and low signal in corresponding ADC map. ADC values of all suspect lesions were determined, but served only as an orientation for characterization of benign/malignant findings. The interpretation of 18F-FDG-PET data, used for differentiating between benign and malignant lesions in PET/MRI and PET/CT ratings was performed qualitatively. A visually increased 18F-FDG uptake in nodal or extra nodal sites higher than in background tissues was considered as an additional sign for involvement with lymphoma [1]. In case of a discrepant finding on PET and MR datasets (e.g. a lesion with unsuspicious morphology and increased focal tracer uptake, or vice versa) the lesions were dedicatedly evaluated in accordance with the criteria used in a previous publication [16]. Therefore, the corresponding PET data were rated superior in PET/MRI and a morphologically unsuspicious lesion with focally elevated 18F-FDG uptake was deemed positive for malignancy.

The tumor stage for each examination was determined in analogy to the revised criteria of the Ann Arbor staging system as proposed by the Lugano classification [1, 17], originally introduced for initial assessment of lymphoma patients. However, the major focus in the present study was to evaluate and demonstrate the overall diagnostic capability of the two imaging modalities to determine disease extent in our patient cohort. Therefore, PET/MRI and MRI examinations for initial staging but also interim scans during treatment, scans for restaging after the end of treatment and for surveillance were analyzed. For the evaluation of the PET-component in PET/CT and PET/MRI the SUVmax and SUVmin of the largest nodal and extra nodal lesions were determined while the number of evaluated lesion was limited to ten per patient.

Finally, a consensus interpretation on a lesion- and patient-basis was performed by two experienced physicians for the determination of the reference standard. Therefore, all 52 PET/CT examinations were analyzed. Additionally, all available histopathological samples as well as results of prior examinations and follow-up imaging (CT, MRI, PET/CT; n = 33, mean duration 239 ± 157 days) were used for the determination of malignant and benign lesions. In accordance with previous publications, lesions that were identified on MRI and/or PET/MRI and could not be identified on PET/CT images, were only included in our ratings if follow-up imaging was available [18]. Conversely, lesions that were identified by PET/CT but missed in DW-MRI or PET/MRI were rated as false-negative.

### Statistical analysis

For statistical analysis the IBM SPSS version 21 software (SPSS Inc, Armonk, NY, USA) was used. Sensitivity, specificity, positive predictive value, negative predictive value and diagnostic accuracy of PET/MRI and MRI for the identification of lymphoma patients were calculated and a McNemar test was used to determine the significance of differences between both ratings. SUVs as well as calculated data of scan duration and radiation exposure are presented as mean values ± standard deviation (SD). Wilcoxon signed-rank test was utilized to indicate potential significant differences between SUVs obtained in PET/MRI and PET/CT. Pearson’s correlation coefficients were calculated and Bland-Altman analyses were performed for determined SUVmax and SUVmean of all PET-positive lymphoma lesions in both hybrid imaging modalities. P-values <0.05 were considered to be statistically significant.

## Results

### PET/MRI vs MRI

**Fig 1:**
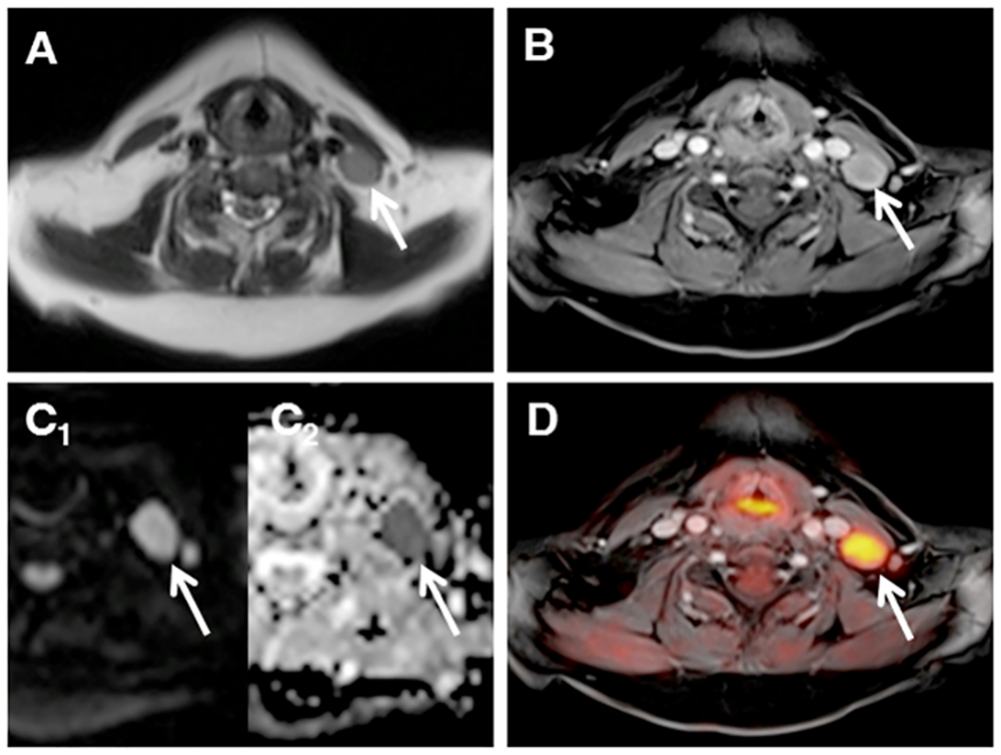
Nodal manifestation of lymphoma (arrows). MR images (T2w Haste, a; T1w VIBE, b) show an enlarged cervical lymph node, which reveals distinctive diffusion restriction (DWI, c_1_; ADC-map, c_2_) as well as pathological glucose metabolism after image fusion in PET/MRI (d).

**Fig 2:**
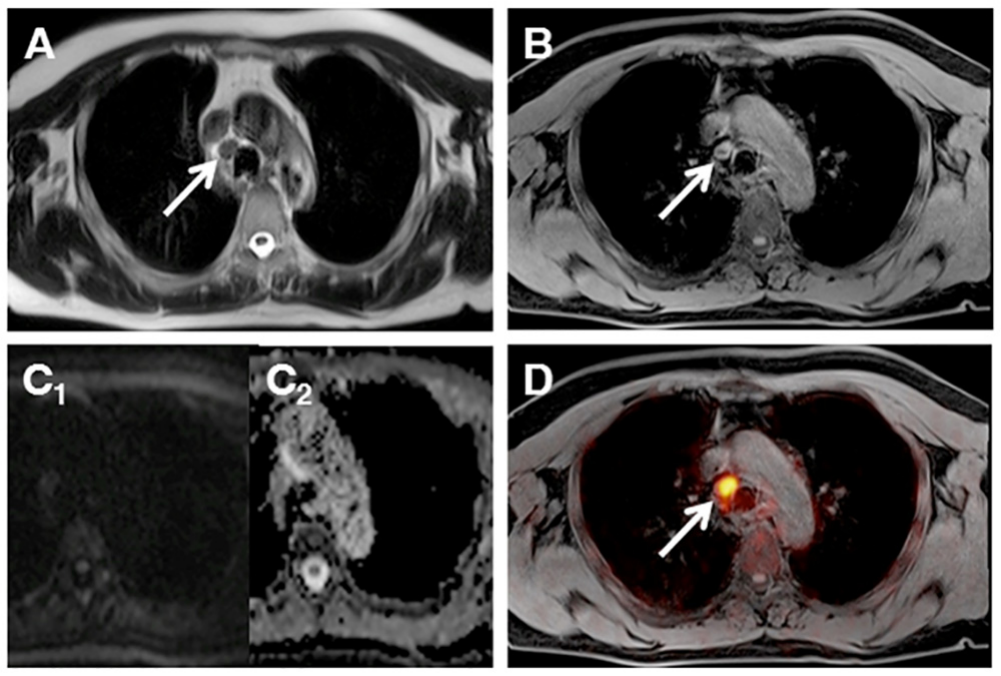
MR images (T2w Haste, a; T1w VIBE, b) show a non-enlarged mediastinal node (arrows), without diffusion restriction (DWI, c1; ADC-map, c2). The identical node shows pathological glucose metabolism and was correctly identified as manifestation of lymphoma with the additional information provided by PET (d).

Based on the reference standard, active lymphoma disease was present in 28 of the 52 whole-body examinations (Table 2, Fig 1). MRI enabled correct identification of active lymphoma in 25 of the 28 (89%) cases, whereas PET/MRI correctly detected disease presence in all 28/28 (100%) examinations. The respective statistical values are shown in Table 3 and did not reveal a significant difference between the two modalities (p>0.05). Furthermore, a total 96 nodal regions were analyzed of which 62 (65%) were affected with active lymphoma disease (Fig 2). Sensitivity, specificity, PPV, NPV and diagnostic accuracy for the identification of involved nodal regions were 84%, 74%, 85%, 71% and 80% with MRI. The respective values with PET/MRI were 97%, 91%, 95%, 94% and 95%. Differences between the two imaging modalities were statistically significant (p<0.05). Additionally, in 13 out of 20 assessed extranodal sites malignant lymphoma was present. Calculated sensitivity, specificity, PPV, NPV and diagnostic accuracy of MRI (92%, 71%, 86%, 83% and 85%) and PET/ MRI (100%, 86%, 93%, 100% and 95%) did not show a significant difference (p>0.05).

**Table 2:** Distribution of investigated nodal regions and extranodal sites.

Localization	Total	Malignant	Benign
Nodal regions	96 (100%)	62 (65%)	34 (35%)
Extranodal sites	20 (100%)	13 (65%)	7 (35%)
▪ Spleen		2 (10%)	
▪ Bone marrow		3 (15%)	1 (5%)
▪ Renal pelvis		1 (5%)	
▪ Liver		1 (5%)	1 (5%)
▪ Soft-tissue		1 (5%)	1 (5%)
▪ Lung		3 (15%)	2 (10%)
▪ Adrenals		1 (5%)	1 (5%)
▪ Muscle		1 (5%)	1 (5%)

**Table 3:** Identification of lymphoma patients in MRI and PET/MRI.

Parameters	MRI	PET/MRI
Sensitivity (95% CI)	89 (72–98)	100 (88–100)
Specificity (95% CI)	83 (63–95)	92 (73–99)
PPV (95% CI)	86 (68–96)	93 (78–99)
NPV (95% CI)	87 (66–97)	100 (85–100)
Accuracy (95% CI)	87 (74–94)	96 (87–100)

Tumor stage was determined in analogy to criteria as proposed by the Lugano classification [1] comprising 19 limited and 8 advanced stage lymphoma manifestations (out of the 28 examinations). In one case, bulky disease was present. MRI enabled correct identification of the tumor stage in 43/52 (83%) examinations. In 6 of the 9 misclassified examinations, MRI overrated the actual tumor stage, based on false-positive findings and underrated the stage in 3 cases due to lymphoma lesions that were falsely interpreted as benign. PET/MRI correctly determined the patients′ disease status in 49/52 (94%) cases and overestimated the actual tumor stage in the remaining 3 examinations. Table 4 summarizes the results of both imaging modalities for the identification of the tumor stage in all 52 examinations.

**Table 4:** Determination of the tumor stage in MRI and PET/MRI, based on the revised staging system.

Tumor stage	Reference	MRI			PET/MRI		
		Correct	Overrated	Underrated	Correct	Overrated	Underrated
Limited	19	14	2	3	18	1	-
Bulky disease	1	1	-	-	1	-	-
Advanced	8	8	-	-	8	-	-
No disease	24	20	4	-	22	2	-
Total	52	43	6	3	49	3	-

### Evaluation of the PET component in PET/CT and PET/MRI

A total of n = 106 18F-FDG-avid lymphoma lesions were analyzed for the comparison of the PET component in PET/CT and PET/MRI.

#### SUV analysis

**Fig 3:**
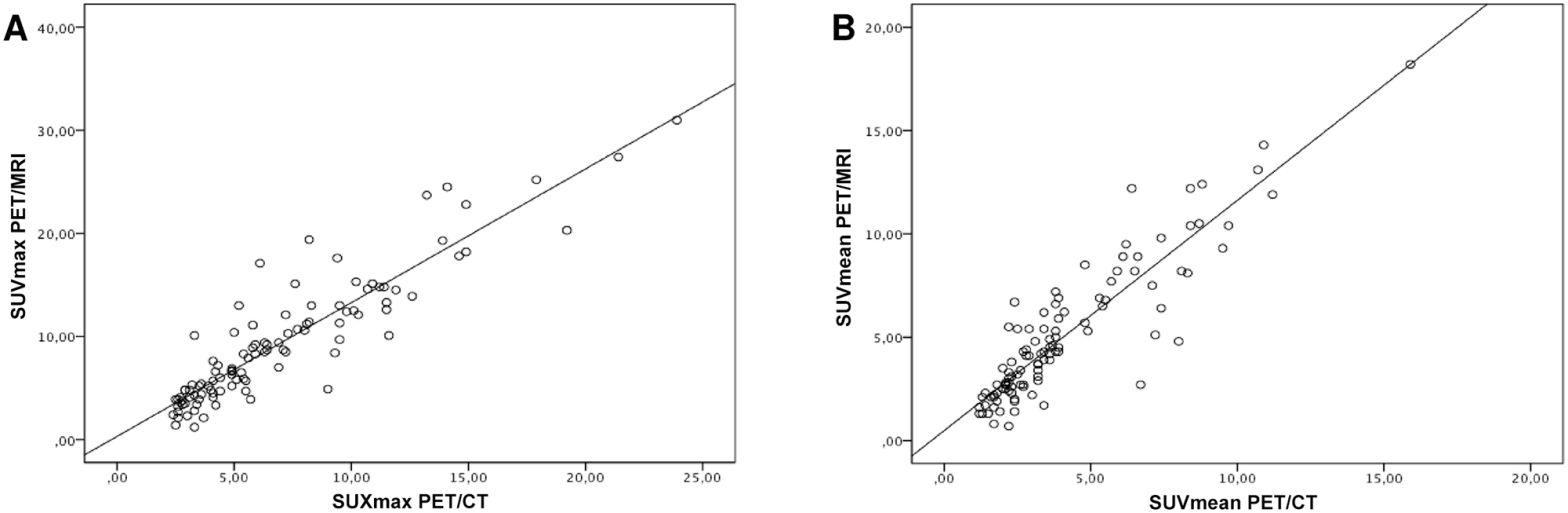
Scatter plots of SUVmax (a) and SUVmean (b) of all PET-positive malignant lesions assessed by PET/CT and PET/MRI.

**Fig 4:**
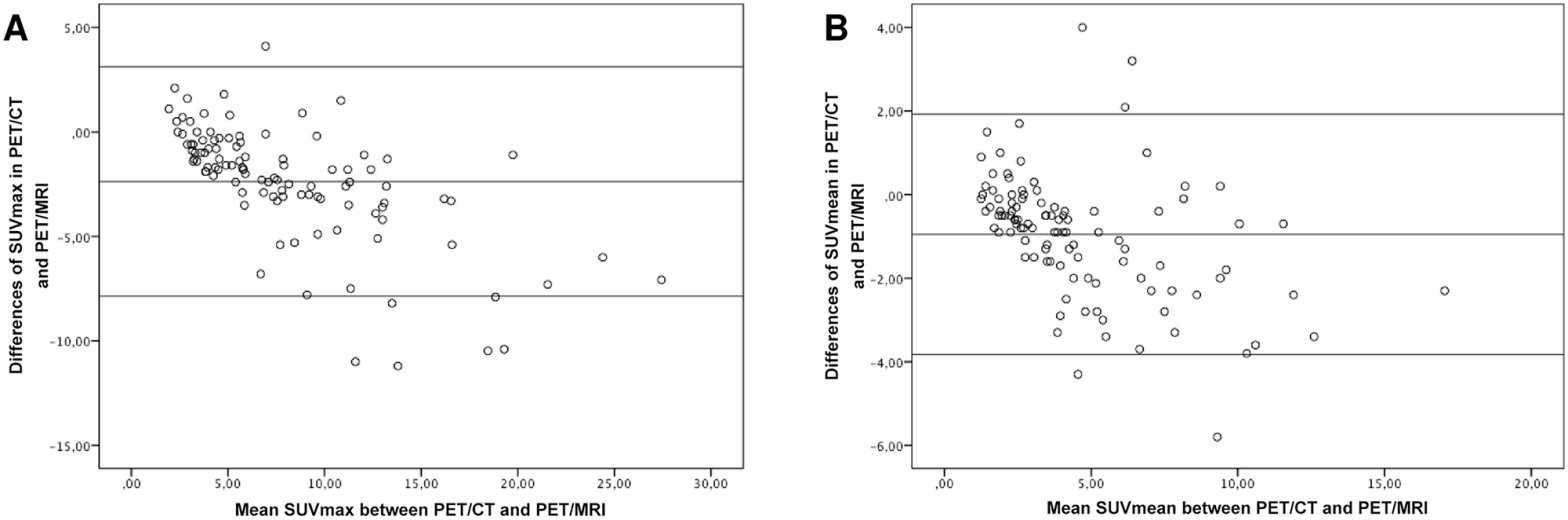
Bland-Altmann plots showing differences of SUVmax (Limits of agreement: 3.12 and -7.86) and SUVmean (Limits of agreement: 1.93 and -3.83) between PET/CT and PET/MRI.

SUVmax and SUVmean of lymphoma lesions was significantly higher in PET/MRI than in PET/CT (SUVmax: 9.3±6.1 vs 6.9±4.3; p<0.05; SUVmean: 5.1±3.3 vs 4.1±2.7; p<0.05). The mean delay between start of PET/CT and start of PET/MRI was 72 min. The SUVmax in both imaging modalities exhibited a strong and positive correlation (R = 0.91; p<0.001, Fig 3a). The SUVmean derived from PET/CT and PET/MRI also revealed a strong and highly significant correlation (R = 0.87; p<0.001, Fig 3b). Bland-Altman analysis was performed to determine lower and upper limits of agreement between PET/CT and PET/MRI for SUVmax (3.12 and -7.86; Fig 4a) and SUVmean (1.93 and -3.83; Fig 4b).

### Estimations of scan duration and radiation exposure

The measured mean duration of whole-body examinations amounted to 17.3±1.9 min for PET/CT and 27.8±3.7 min for PET/MRI.

Mean effective dose of all whole-body PET/CT examinations amounted to 14.6±8.7 mSv, with PET accounting for 5.2±1.0 mSv (35.6%). Calculated radiation dose was 23.2±4.8 mSv for a full-dose PET/CT examinations and 7.2±1.3 mSv for all low-dose PET/CT scans. Radiation dose of the corresponding whole-body CT scan amounted to 17.9±5.3 mSv (77.2%) in a full-dose PET/CT and 2.1±0,7 mSv (29.2%) in a low-dose scan, while the proportions of administered 18F-FDG in PET was 5.3±0.9 mSv (full-dose PET/CT, 22.8%) and 5.1±1.1 mSv (low-dose PET/CT, 70.8%), respectively.

## Discussion

The present study investigated the diagnostic potential of a fast protocol for integrated PET/MRI used for dedicated tumor staging of patients with lymphoma. Combining simultaneously obtained PET and MRI data for image interpretation enabled a significantly better diagnostic performance for the assessment of nodal manifestations of lymphoma if compared to MRI alone. In addition, disease status based on the revised staging criteria was correctly identified in a higher number of patients using PET/MRI.

Within the last years, hybrid imaging, in terms of PET/CT has been well established as a high quality imaging tool for lymphoma diagnostics, integrating high resolution anatomical and metabolic information [3, 4]. Adding the additional information provided by 18F-FDG PET to CT enables a higher detectability of active lymphoma lesions and facilitates therapy response assessment even in cases, in which structural changes have not yet become visible [19–21]. Therefore, PET/CT has been proven highly valuable for a diagnostic workup of FDG-avid lymphoma subtypes, yet also considering the relatively high radiation exposure, mainly caused by the CT-component [9, 10].

**Fig 5:**
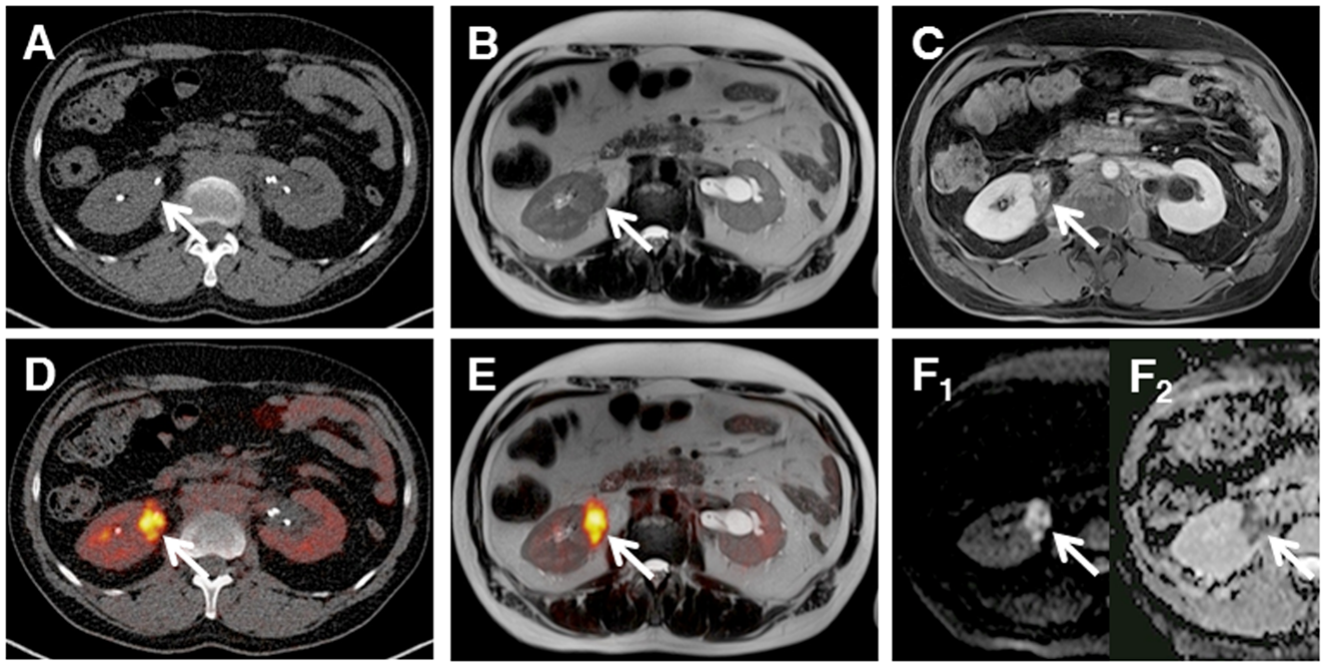
Manifestation of Lymphoma within the renal pelvis (arrows), which is not clearly visible on low-dose CT images (a), but can be identified on MR images (T2w Haste ax., b; T1w VIBE ax. post-contrast, c). The identical lesion reveals pathological glucose metabolism after image fusion on PET/CT (d) and PET/MRI (e) as well as high signal in DWI (f_1_) and a signal drop on the ADC-map (f_2_).

The recent introduction of integrated PET/MRI systems may represent a promising alternative to PET/CT, having demonstrated its high and comparable diagnostic capacity to PET/CT in numerous publications [22, 23]. Besides an extension of examination time due to the interchange of the morphological part from CT to MRI, PET/MRI enables a remarkable reduction of ionizing radiation dose of about 73 to 77% per examination [9, 10]. Our results strengthen these findings, demonstrating an overall reduction of radiation exposure of about two thirds by using PET/MRI as an alternative to PET/CT (low-dose and full-dose). Especially lymphoma patients might particularly benefit from this new imaging modality, considering the percentage of a younger patient population and the need for repetitive examinations, increasing the risk of radiation associated second malignancies [24, 25]. Moreover, even if radiation savings compared to low-dose PET/CT scans are limited (29%), PET/MRI offers high-quality morphologic information in addition to the PET data (Fig 5), which enables a better characterization of suspicious findings. In addition, fast-PET/MRI provides a high quality diagnostic performance within an appropriate scan duration, exceeding the average scan duration of a whole-body PET/CT for only about 10 minutes.

An initial study by Platzek and colleagues reported a high sensitivity and specificity of PET/MRI for the detection of lymphomas in a region-based analysis [12]. Our results support these findings, yielding a correct identification of all patients with viable lymphomas using PET/MRI, while two patients without evidence of malignancy were rated false-positive. Those misinterpretations occurred due to a focally increased tracer uptake of nodal lesions, which may have been caused by the subsequent acquisition of PET/MRI datasets with an average delay of 72 min after PET/CT. Previous studies could already show altered distributions of 18F-FDG in certain tissues and organs on PET/MRI datasets, which were acquired about one hour after PET/CT [26, 27].

The successful introduction of diffusion-weighted imaging as an additional functional parameter to morphological MR imaging enabled an increase in diagnostic accuracy for the identification and characterization of tumor lesions [28]. Some studies reported promising results of MRI with DWI in staging of lymphoma patients, which were only slightly inferior to that from PET/CT [29, 30]. One recently published work by Heacock and colleagues compared the diagnostic ability of MRI and PET/MRI and showed a higher staging performance using PET/MRI [11]. These findings go in line with our results, demonstrating a significantly better performance of PET/MRI for the detection of lymphomas as well as for the determination of the correct tumor stage. In accordance with previous publications, MRI revealed a tendency to overrate the actual tumor stage, with substantial consequences on further patient management [31, 32]. However, staging indolent, frequently non-FDG-avid lymphoma subtypes, the use of MRI including DWI might be highly beneficial and potentially superior if compared to PET imaging as it has been shown in a recently published study by Giraudo and colleagues [33].

The use of PET has been recommended as an integral part for staging and clinical evaluation of 18F-FDG-avid lymphomas, representing the vast majority of lymphoma types in our study [1]. Besides visual assessment, quantitative SUV measurements are commonly performed to assess viable lymphoma lesions as well as for the determination of therapeutic response due to metabolic changes under therapy [7]. One major challenge and general point of discussion when comparing PET/MR and PET/CT hybrid imaging lies in physical differences of MR- and CT-based attenuation correction, potentially leading to differences in absolute SUV measurements between both hybrid imaging modalities. Investigating the applicability of the PET_PET/MR_-component for the evaluation of lymphoma patients, our results reveal a strong positive correlation between SUVs obtained from PET/CT and PET/MRI. A number of previously published studies support these results, showing a high correlation of the SUVs acquired in both imaging modalities for parenchymatous organs as well as for different tumor types [26, 34]. Accordingly, these data underline the validity of SUVs derived from PET/MRI datasets for the use in oncological imaging. While most previous publications show an overall increase of SUVmax (in different tumor entities, including lymphoma), a recent publication by Heacock et al. revealed an overall decrease of the SUVmax on subsequently acquired PET/(MRI) data [11]. Our results go in line with most publications, demonstrating significantly higher values for SUVmax and SUVmean on PET/MRI datasets which were obtained with a one hour delay after a PET/CT [35]. This might be explained by an increasing 18F-FDG accumulation in malignant cells within the period of prolonged tracer uptake after intravenous administration.

Our study is not free of limitations. First, the patient cohort consisted of different lymphoma subtypes (Table 1). Therefore, subgroup analyses would have been desirable, yet, would not have been reasonable due to limited patient numbers. Accordingly, these preliminary results should be confirmed in future studies investigating the staging performance of PET/MRI for different lymphoma types. Second, the majority of patients revealed 18F-FDG-avid lymphomas, which justifies the use of PET/CT as the main part of the standard of reference, hence restricting a direct comparison of PET/MRI and PET/CT in the evaluation of lymphoma patients. Another limitation lies in the restricted reference standard, mainly caused by the unavailability of histopathological confirmation of all suspicious lesions. Therefore, in accordance with previous publications, we used all applicable information in terms of the results from PET/CT imaging as well as all available histopathological results and cross-sectional imaging follow-up as reference standard [36]. Finally, we used two different protocols (low-dose or full-dose examinations) for PET/CT imaging. A concordant protocol would have been desirable, yet, the study set up reflects clinical staging algorithms. Accordingly, in low-dose PET/CT examinations morphological criteria for the formation of the reference standard have been limited, which might have affected the determination of malignant of benign lesions.

## Conclusion

The present study demonstrates the high diagnostic value of a fast protocol for integrated PET/MRI for staging lymphoma patients, enabling high quality assessment of morphologic and metabolic data while maintaining comparable examinations times with markedly reduced radiation exposure when compared to PET/CT. Furthermore, our results demonstrate the usefulness of 18F-FDG PET data as a valuable additive to MR imaging for a more accurate evaluation and tumor staging of lymphoma patients.
